# Reduced vagal control of the heart in high-fat diet mice: a potential role of increased butyrylcholinesterase

**DOI:** 10.14814/phy2.12609

**Published:** 2015-11-04

**Authors:** Sigurd Hartnett, Hongbo Gao, Sabrina Schnack, Yifan Li

**Affiliations:** Division of Basic Biomedical Sciences, Sanford School of Medicine, University of South DakotaVermillion, South Dakota

**Keywords:** Butyrylcholinesterase, heart rate, mice, obesity, parasympathetic

## Abstract

Suppressed parasympathetic function is commonly present in cardiovascular diseases, aging, obesity, and various other health conditions. Impaired parasympathetic action is known as a detrimental factor and contributes to the adverse outcomes in these conditions. However, the underlying mechanisms remain to be fully addressed. In this study, using high-fat diet (HFD)-induced obese mice as a model, the potential peripheral mechanisms underlying the impaired parasympathetic vagal control of the heart was examined. The HFD induced obesity and metabolic disorder in mice. These obese mice exhibited an attenuated response in heart rate to vagal stimulation, indicating impairment of peripheral parasympathetic activity in the heart. In cholinergic function-related proteins in the atria, protein levels of choline transporter and vesicular acetylcholine transporter were not decreased but increased, and type 2 muscarinic receptors showed a trend toward a reduction in HFD mice atria as compared with regular diet (RD) mice controls. While the protein level of acetylcholinesterase was not different, butyrylcholinesterase (BChE) protein level showed a twofold increase in HFD mice atria as compared with RD mice. Functionally, inhibition of BChE activity partially and significantly improved the attenuated response in heart rate to vagal stimulation in HFD mice. Collectively, these data suggest that increased BChE activity in the atria may contribute to the decreased parasympathetic function in HFD-induced obese mice.

## Introduction

The activities of autonomic nervous systems are often altered in various cardiovascular diseases (Brook and Julius 2000), aging (Corbett et al. 2007; De Meersman and Stein 2007), and obesity (Alvarez et al. 2002), and characterized as elevated sympathetic nervous system (SNS) activity and suppressed parasympathetic nervous system (PSNS) activity. These imbalanced autonomic functions elicit detrimental effects on the heart in these health conditions (Malpas 2010). While sympathetic over excitation has been extensively studied and has been targeted therapeutically in various cardiovascular diseases, the suppressed parasympathetic function has not been paid a great deal of attention until recently (Olshansky et al. 2008). PSNS suppression and withdrawal has long been recognized as a contributor to increased cardiac events, sudden death, and poor clinical outcomes in patients with heart diseases (La Rovere et al. 1998; Thayer and Lane 2007; Olshansky et al. 2008; Thayer et al. 2010). Yet, the mechanisms by which PSNS is suppressed are still poorly understood and effective therapeutic means to restore PSNS function in cardiovascular diseases are lacking.

Obesity is an increasing health issue in the United States and worldwide. It was estimated in 2009–2010 that 35% of U.S. adults were obese (Ogden et al. 2012). Obesity is an independent risk factor for cardiovascular diseases, the leading cause of mortality in the United States (Mozaffarian et al. 2014). Obesity increases the risks of arrhythmia (Wang et al. 2004), myocardial infarction (Yusuf et al. 2005), heart failure (Guglin et al. 2014), and hypertension (Hall et al. 2010; Landsberg et al. 2013). The pathogenic factors that arise from obesity include altered metabolic profiles, increased cardiac workload, oxidative stress, and inflammation. Obesity also causes altered autonomic activity, including suppressed parasympathetic nervous system (PSNS) activity (Grassi et al. 1995; Huggett et al. 2004; Rodriguez-Colon et al. 2011), which is an additional cardiovascular hostile change.

A high-fat diet (HFD) has been found to reduce parasympathetic activity in rats (Afonso et al. 2010). In this study, we examined parasympathetic vagal control of the heart in HFD-induced obese mice. While both central and peripheral mechanisms of impaired PSNS functions have been evident (Bibevski and Dunlap 1999, 2004; Wang et al. 2001), here we focused on the peripheral PSNS function by examining the heart rate response to vagal stimulation and proteins that are related to acetylcholine synthesis, release, and breakdown in atrial tissues.

## Methods

### Animal model

Adult male and female ICR mice at 6–8 weeks of age and body weight around 22–26 g were purchased from Harlan Laboratories, Inc. (Indianapolis, IN). All protocols using animals in this report were reviewed and approved by the Institutional Animal Care and Use Committee of the University of South Dakota and were in compliance with *Guide for the Care and Use of Laboratory Animals*. The mice were grouped and fed either a regular diet (RD) or high-fat diet (HFD) for 14 weeks. RD is Harlan global diet 2020X (Harlan Laboratories), which has energy density of 3.1 kcal/g and 16% of total calories from fat. HFD is TD06414 (Harlan Laboratories), which has energy density of 5.1 kcal/g and 60% of total calories from fat. During each diet regimen, the food intake and body weight of animals were weighed weekly. Body weight gain for each mouse was calculated by subtracting initial body weight at the beginning of the diet regimen from body weight at each week. Nonfasting blood glucose levels were measured every other week using a commercially available blood glucose monitor (One Touch Ultra 2 Meter) from a drop of blood drawn from a small incision in the tail.

### Glucose and insulin tolerance testing

The glucose tolerance test was conducted at week 13. Mice were fasted overnight, later injected with d-glucose (1 g/kg, i.p.), and blood glucose (mg/dL) was measured at 0, 15, 30, 60, 90, and 120 min. Mice were allowed to recover for 1 week. At week 14, the insulin tolerance test was conducted. Mice were fasted for approximately 4 h and injected with insulin (0.75 *μ*g/kg, i.p.). The procedure and timing for measuring blood glucose were the same as performed for the glucose tolerance test.

### Electrocardiograph and vagus nerve stimulation

At the end of the diet regimens, mice were anesthetized with a combination of urethane (2 g/kg) and *α*-chloralose (50 mg/kg, i.p.) in normal saline and placed in a supine position on a warming pad. A pair of electrodes were subcutaneously placed on the right forelimb and left hind limb to record Lead II surface electrocardiography (ECG).

In the anesthetized state, the right cervical vagus nerve was carefully isolated with the rostral end being severed and the caudal end remaining intact. The nerve was placed on a bipolar electrode and covered with mixed mineral oil and Vaseline to prevent drying. Following stabilization for 5 min, the vague nerve was stimulated with rectangular electrical pulses at 3 V, frequencies from 2 to 12 Hz, and a duration of 2 msec. The signals of stimuli, ECG, and changes in heart rate were recorded by Powerlab data system (Model 8/sp, ADInstruments Inc.).

### Pharmacologic treatments

To examine the contribution of butyrylcholinesterase to parasympathetic nervous system suppression in obesity, HFD and RD mice were intraperitoneally injected with a specific butyrylcholinesterase inhibitor bambuterol (0.05 mg/kg) (Kovarik et al. 1999) or saline following baseline vagal stimulation. Responses to vagal stimulation were recorded 10 min post injection.

### Tissue/blood collection

At the end of diet regimens, under anesthesia, mice were sacrificed by cervical dislocation. Blood was collected, centrifuged, and the resulting serum was stored at −80°C. Tissues including the heart were collected, rapidly frozen on dry ice, and stored at −80°C. Abdominal fat was also weighed.

### Western blot analysis

The right atrium, which is highly innervated by the PSNS, was lysed in RIPA buffer containing protease inhibitor cocktail and 0.1% SDS. Following centrifugation at 10,000 *g* for 5 min, supernatant was collected and protein concentration was assessed and normalized. Mixed with loading buffer containing beta-mercaptoethanol (BME) and boiled, the protein sample was subjected to standard SDS-PAGE and transfer procedures as described previously (Freeling et al. 2008; LaCroix et al. 2008). Membranes were immunoblotted using the following primary antibodies: rabbit anticholine transporter (CHT, a custom antibody (Ferguson et al. 2003) produced by Genemed Synthesis, San Antonio, TX), rabbit antibutyrylcholinesterase (BChE, Sigma-Aldrich, St. Louis, MO), rabbit antiacetylcholinesterase (AChE), rabbit antimuscarinic type 2 receptor (M2AChR), rabbit antivesicular acetylcholine transporter (VAChT, Santa Cruz Inc., Santa Cruz, CA), rabbit anti-*β*2 adrenergic receptor (*β*2AR, Abcam Inc., Cambridge, MA), and rabbit antiactin (Santa Cruz Inc.). Following the use of an appropriate fluorescently conjugated secondary antibody, membranes were imaged using LI-COR Imager (LICOR Biosciences, Lincoln, NE), and quantified using Image Studio (LICOR Biosciences) or ImageJ (NIH) software. Actin was used as a loading control.

### Biochemical assays

Serum levels of cholesterol, glucose (Life Technologies, Inc., Grand Island, NY), and resistin (R&D Systems, Inc., Minneapolis, MN) were detected using commercial kits and read by a Tecan Microplate Reader (Tecan US Inc., Morrisville, NC). The activity of serum BChE was measured by a colorimetric assay using butyrylthiocholine iodide (BTC), a specific BChE substrate, and 5,5′-dithiobis (2-nitrobenzoic acid) (DTNB) as a color indicator, as previously reported (Naik et al. 2013). The optical density (OD) of samples was read by the plate reader immediately after the addition of BTC, which was considered the background signal, and again 30 min later. For calculating the relative BChE activity rate, the difference between the OD values at 30 and 0 min was divided by 30 min.

### Data analysis

Raw data and descriptive statistics were calculated using Microsoft Excel. Data are presented as mean ± standard error of the mean (SEM). Many inferential statistical methods were used to analyze the data including one- or two-factor analysis of variance (ANOVA) and unpaired two-tailed Student’s *t*-test, where appropriate. As most physiological data included repeated vagal nerve stimulation at pre- and postinjection time points in the same mouse, two-factor repeated measure ANOVA was utilized. All ANOVA calculations were followed by either Tukey or Holm–Sidak post hoc analyses. Western blots were quantified using Image Studio (LICOR) or Image J (NIH) software. Statistical analyses were conducted using GraphPad Prism Statistical Software version 6.05 (San Diego, CA).

## Results

### HFD-induced metabolic disorder

**Figure 1:**
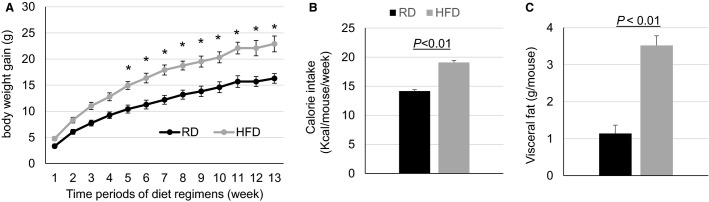
HFD-induced obesity in mice. Weekly (A) body weight gains (RD *n* = 18, HFD *n* = 24) and (B) calorie intake (RD *n* = 37, HFD *n* = 50) per mouse; and (C) average visceral fat at week 14 of diet regimens (RD *n* = 14, HFD *n* = 19). Black curve and bars are RD and gray curve and bars are HFD. *P < 0.01 versus RD.

**Figure 2:**
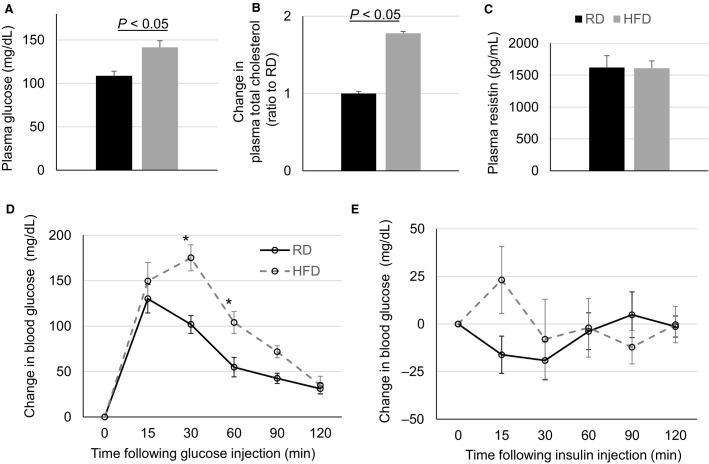
Metabolic alterations in HFD mice. (A) Nonfasting blood glucose levels at week 11 of diet regimen (RD *n* = 6, HFD *n* = 6). (B) Change in blood total cholesterol levels at week 9 of diet regimen (RD *n* = 6, HFD *n* = 6). (C) Blood resistin levels at week 14 of diet regimen (RD *n* = 5, HFD *n* = 10). (D) Glucose tolerance test at week 13 of diet regimen (RD *n* = 6, HFD *n* = 6). (E) Insulin tolerance test at week 14 of diet regimen (RD *n* = 6, HFD *n* = 6). In all figures, black curves and bars are RD and gray curves and bars are HFD. *P <* *0.05 versus RD.

Over the diet regimens, mice fed HFD consumed more total calories, gained more body weight (reaching 42–56 g in HFD compared with 40–45 g in RD at the end of the diet regimen), and had greater amounts of visceral fat, as compared with RD mice (Fig.1). These changes were constant in both male and female mice. Moreover, the HFD mice exhibit significantly increased plasma total cholesterol and nonfasting blood glucose levels as compared with RD mice (Fig.2). Glucose tolerance test response and insulin tolerance test response were abnormal in HFD mice as compared to the RD mice (Fig.2). However, plasma resistin level was unchanged in HFD mice as compared with RD mice. Overall, these results show that the HFD caused obesity, metabolic disorder, and insulin resistance in mice.

### Reduced vagal control of the heart in HFD mice

**Figure 3:**
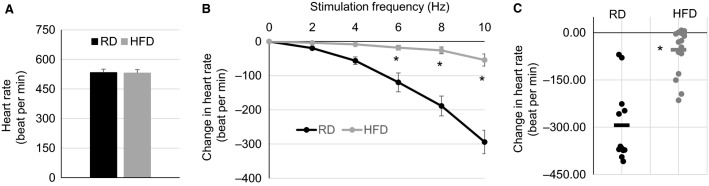
Unchanged heart rate baseline and reduced response to vagal stimulation at week 14 of diet regimens. (A) Basal level of heart rate in anesthetized mice fed RD or HFD (RD *n* = 12, HFD *n* = 17). (B) Changes in heart rate in responses to vagal stimulation (RD *n* = 12, HFD *n* = 17). (C) Individual response in heart rate to vagal stimulation at 10 Hz (RD *n* = 12, HFD *n* = 17). In all figures, black curve, bar, and dots are RD and gray curve, bar, and dots are HFD. *P <* *0.01 versus RD.

To assess the impact of obesity and metabolic disorder on PSNS control of the heart, the responses in heart rate to vagal stimulation were tested in both male and female mice at the end of diet regimens. Figure3B and C are the combined quantifications of heart rate responses to vagal stimulation in both genders at 14 weeks of RD or HFD, showing a significant reduction of bradycardic responses induced by vagal stimulation in HFD mice as compared with RD mice. In a separate analysis (data not shown), male and female mice exhibited similar reduction of bradycardic responses to vagal stimulation at 14 weeks on HFD regimens as compared with RD. These results suggest that the function of the vagal parasympathetic innervation within the heart is impaired in the HFD-induced obese mice. However, the basal level of heart rate was not different between HFD and RD mice (Fig.3A), suggesting the tonic parasympathetic effect on the heart may not be significantly changed.

### Changes in cholinergic function-related proteins in atria

**Figure 4:**
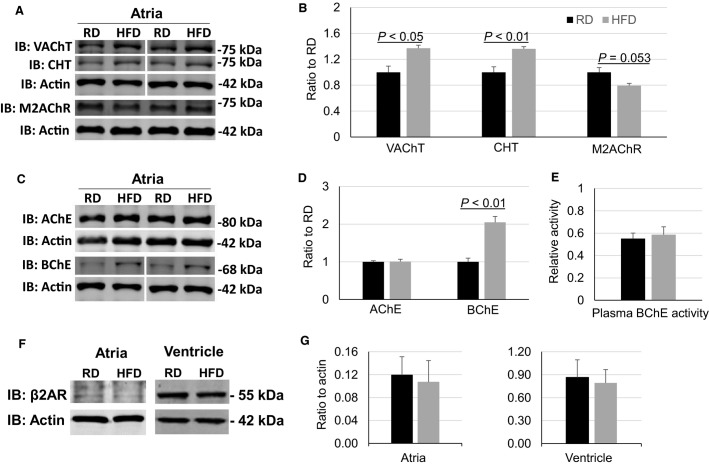
Changes in major cholinergic proteins and *β*2AR in atria. (A, B) Western blot images and quantifications of VAChT, CHT, and M2AChR protein levels in atria from RD and HFD mice (RD *n* = 5, HFD *n* = 4). (C, D) Western blot images and quantifications of AChE and BChE protein levels in atria from RD and HFD mice (RD *n* = 5, HFD *n* = 4). (E) Relative plasma BChE activity (RD *n* = 6, HFD *n* = 6). (F, G) Western blot images and quantifications of *β*2AR in atrial and ventricular tissues (atrial *n* = 3 and ventricular *n* = 4). In all figures, black bars are RD and gray bars are HFD.

PSNS ganglia and postganglionic terminals use acetylcholine (Ach) as their primary neurotransmitter. Thus, we explored the possible alterations of proteins related to Ach synthesis, release, and breakdown in the right atrium, which is the primary site that receives PSNS innervation and controls heart rate. Choline transporter (CHT) is known as the rate-limiting protein for Ach synthesis (Black and Rylett 2012), and vesicular Ach transporter (VAChT) is critical for Ach packing and release (de Castro et al. 2009). Unexpectedly, the protein levels of both CHT and VAChT were significantly increased instead of decreased in atrial tissues from HFD mice as compared with RD mice (Fig.4A,B). Given the reduced PSNS control of heart rate, these increases in CHT and VAChT protein levels may reflect compensatory responses. Muscarinic type 2 Ach receptors (M2AChR) are the major receptors that mediate PSNS regulation of the heart. The protein level of M2AChR exhibited a trend toward a reduction in atria of HFD mice but did not reach statistical significance as compared with RD mice.

Ach breakdown and inactivation in cholinergic synapses is primarily conducted by acetylcholinesterase (AChE). However, there was no difference in the atrial protein level of AChE between HFD and RD mice. In contrast, however, the protein level of butyrylcholinesterase (BChE) had approximately a twofold increase in atrial tissues from HFD mice as compared to RD mice (Fig.4C and D). Additionally, BChE activity in plasma was not increased in HFD mice as compared with RD mice (Fig.4E), suggesting the increased BChE by HFD may be tissue specific or occur earlier in tissues.

The protein level of *β*2AR was very low in atrial tissues as compared with that in ventricular tissues. Although there was a trend of reduction in HFD, *β*2AR proteins in atrial and ventricular tissues were not significantly different between HFD and RD mice (Fig. 4F and G).

### Effect of BChE activity inhibition on PSNS function

**Figure 5:**
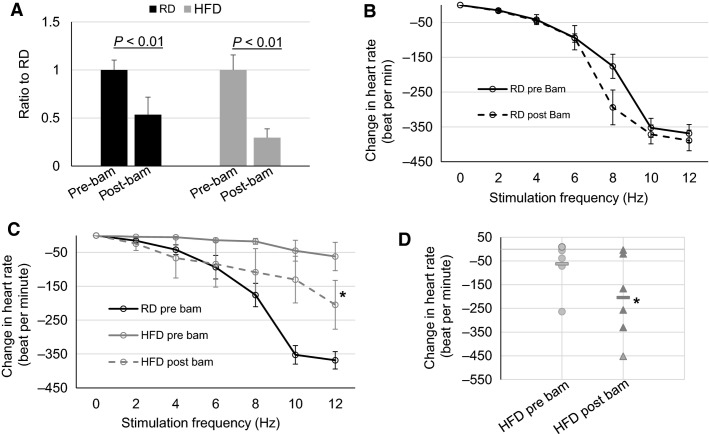
Effect of BChE inhibition on responses to vagal stimulation at week 14 of diet regimen. (A) Relative plasma BChE activities pre- and postadministration of the BChE inhibitor, bambuterol (RD *n* = 5, HFD *n* = 6). (B) Heart rate responses to vagal stimulation in RD mice pre- and postbambuterol administration (*n* = 5). (C) Heart rate responses to vagal stimulation pre- and postbambuterol administration in HFD mice (*n* = 6), in contrast to the response of the prebambuterol in RD mice (*n* = 5). In all above figures, black curve and bars are RD and gray curves and bar are HFD; solid curves are prebambuterol and dash curves are postbambuterol. (D) Individual responses at 12 Hz stimulation of HFD prebambuterol (●, *n* = 6) or HFD postbambuterol (▲, *n* = 6). “▬” represent mean value of the group. *P <* *0.05 versus HFD prebambuterol.

It is generally believed that normally BChE has no significant effect on Ach breakdown in cholinergic synapses (Johnson and Moore 2012). However, whether the increased BChE protein levels led to enhanced ACh breakdown and reduced cholinergic function are unknown. Thus, we tested whether the elevated BChE level may have any role in the reduced vagal control of heart rate in HFD mice. Bambuterol’s inhibitory effect on BChE activity has been well documented (Sinko et al. 2005; Bosak et al. 2008). Because bambuterol is also a beta 2 adrenergic receptor (*β*2AR) agonist, we assessed the protein level of *β*2AR in atrial tissues, which was low and unchanged in HFD, as mentioned above. To verify the inhibitory effect of bambuterol on BChE, serum BChE activity was assessed before and after the intraperitoneal injection of bambuterol. Ten minutes postbambuterol administration, the serum BChE activity was significantly reduced as compared with that before bambuterol injection (Fig.5A), suggesting the effectiveness of bambuterol in inhibiting BChE activity.

Functionally, the responses in heart rate to vagal stimulation showed similar changes before and after the injection of bambuterol in RD mice (Fig.5B). These data support the notion that BChE has no significant effect on cholinergic function in the normal condition. In contrast, in HFD mice 10 min after the injection with bambuterol, the vagal stimulation-induced bradycardiac response was significantly enhanced as compared with the responses before bambuterol administration (saline control); yet, it was still significantly lower than the responses in RD mice (Fig.5C), suggesting the administration of bambuterol partially restored the PSNS impairment observed in the HFD mice. Given the increased BChE protein level in HFD atria, these data suggest that the increased BChE level in atria may contribute to the suppressed PSNS cholinergic function in atria in HFD mice.

## Discussion

Impairments of PSNS function are present in various cardiovascular diseases, aging, and obesity, and are associated with increased mortality and morbidity in these conditions; yet, the underlying mechanisms remain to be fully understood (Olshansky et al. 2008). The present study provides novel evidence that upregulated BChE may be involved in the reduced PSNS function in HFD-induced obese mice and inhibition of BChE activity partially restores the PSNS control of the heart rate.

Regarding the possible causes of PSNS impairments in diseases conditions, both central (Tomaszek et al. 2013) and peripheral (Bibevski and Dunlap 1999) mechanisms have been evidenced. In the periphery, it has been found that reduced parasympathetic control of the heart in chronic heart failure involved impaired cholinergic transmission at parasympathetic cardiac ganglia (Bibevski and Dunlap 1999). Whether the peripheral mechanisms of impaired PSNS function are also present in other diseases conditions are unknown. In this study, we examined the possible peripheral parasympathetic dysfunction in HFD obese mice by observing the heart rate responses to right vagus nerve stimulation when the rostral end is severed and caudal end remains intact. As the data showed, vagal stimulation-induced, frequency-dependent bradycardia was significantly attenuated in HFD mice as compared with RD mice, which clearly indicates the impaired peripheral PSNS function in the HFD obese mice.

Ach is the primary neurotransmitter at both PSNS ganglionic synapses and postganglionic nerve terminals. We then analyzed the major cholinergic transmission-related proteins in the right atria, which is the primary site of PSNS innervation in the heart and regulation of heart rate. The protein level of M2AChR, the major cholinergic receptors that mediate PSNS regulation of the heart rate, showed a trend toward a reduction, yet did not reach significance, in HFD mice as compared with RD mice. The protein levels of CHT and VAChT, two critical transporters that are responsible for Ach synthesis and release, were surprisingly increased in HFD atria. Given the reduced PSNS function to the atria, the increases of these proteins possibly represent a compensatory response in attempt to restore the declined parasympathetic function. Taken together, these data do not suggest the possibility that impaired presynaptic Ach synthesis and release, or the postsynaptic receptor transduction are the major causes of the impaired peripheral PSNS function in this model.

In cholinergic synapses, released Ach is quickly degraded and deactivated by cholinesterases, primarily acetylcholine esterase (AChE). We then determined whether the Ach breakdown and deactivation were enhanced in HFD mice. There was no change in protein level of the primary Ach hydrolase AChE in HFD mice atria compared with RD mice. Unexpectedly, however, the protein level of butyrylcholinesterase (BChE) was significantly increased in HFD mice atria as compared to RD mice. Importantly, functional testing further revealed that inhibition of BChE activity by the administration of bambuterol partially but significantly improved the heart rate response to vagal stimulation in HFD mice as compared with the responses in HFD mice prior to the bambuterol treatment. Together, these data suggest that the increased BChE may, at least partially, contribute to the peripheral PSNS impairment in this model.

This finding is surprising and novel because BChE normally has minimal effect on breakdown of Ach and cholinergic synaptic functions. BChE is less efficient in hydrolyzing Ach and less abundant in cholinergic synapses than AChE (Duysen et al. 2009; Johnson and Moore 2012). Mice with BChE genetic depletion have normal cholinergic function (Duysen et al. 2009). Consistent with this, our data show that inhibition of BChE by administration with bambuterol did not affect the baseline heart rate and the response to vagal stimulation in RD mice, confirming the minimal role of BChE in PSNS function in the normal condition. It is not clear to date, however, whether BChE function is enhanced and thus becomes more significant in Ach clearance as the enzyme level is upregulated and more abundant during particular conditions. Our data provide novel evidence that suggests this possibility.

At this time, the mechanisms upregulating BChE and its overall functional significance in HFD mice is not clear. In fact, the precise biological function of BChE is still poorly understood. This enzyme has been considered functionally redundant (Johnson and Moore 2012). BChE knockout mice are healthy (Duysen et al. 2009). However, there is evidence suggesting that BChE may elicit detoxification in the body. For example, BChE knockout mice have increased susceptibility to cocaine toxicity (Duysen et al. 2008, 2009). Interestingly, these BChE knockout mice also seem to be more susceptible to a high-fat diet and become more obese than the wild type control upon being fed HFD (Li et al. 2008). According to these observations, one plausible hypothesis is that the upregulation of BChE may be a compensatory response to HFD-induced metabolic challenges and may play some role in regulating lipid metabolism and certain adverse factors during metabolic disturbances in the body. As a “side effect,” however, this increased BChE may result in the enhanced Ach degradation and consequently attenuated cholinergic functions, such as the function at parasympathetic peripheral terminals. In our study, the serum BChE activity was not significantly increased in HFD mice, suggesting the increased BChE may be a tissue-specific change, at least within the 14 weeks of HFD used in this study. In fact, increased BChE has been reported in obesity and diabetes patients and the enzyme has been proposed as a biomarker for obesity and metabolic disorder (Iwasaki et al. 2007; De Bona et al. 2013; Sato et al. 2014), further suggesting the BChE may be involved in some compensatory mechanisms in response to metabolic challenges.

Our data showed that administration of bambuterol partially but significantly restored the attenuated bradycardiac response to vagal stimulation in HFD mice. Bambuterol is known as an adrenergic beta 2 receptor (*β*2AR) agonist and a BChE inhibitor (Sinko et al. 2005). Based on our observation, it is unlikely that the restoration of PSNS control of heart rate in HFD mice is due to its *β*2AR agonist effect. First, *β*2AR activation increases heart rate but at the dosage used in this study, bambuterol did not alter the baseline heart rate in either RD or HFD mice. Second, *β*2AR activation inhibits rather than facilitates PSNS action in the heart. Third, administration of bambuterol effectively inhibits BChE activity. Fourth, our data showed that *β*2AR protein level was very low in atria as compared with that in ventricular tissues and the protein level of the receptor was unchanged in HFD mice as compared with RD mice. Finally, bambuterol only positively affected the reduced response to vagal stimulation in HFD mice that had an increase in BChE but had no effect on RD mice. Therefore, the improved PSNS response in HFD mice by bambuterol suggest a potential role of increased BChE in the impaired PSNS dysfunction in this model. In addition, this result also suggests a potentially clinically practicable means to treat PSNS dysfunction. Bambuterol is clinically used as a bronchodilator for treatments of asthma (Sitar et al. 1993) and COPD (Cazzola et al. 1999) and thus could be easily repurposed for restoring PSNS function, should this effect reported here be confirmed in humans. Given the fact that PSNS dysfunction is widely present and is a risk factor in various cardiovascular diseases, aging, and obesity, this drug and other BChE inhibitors may offer new opportunity to improve PSNS function in these diseases.

While this study provides novel and exciting evidence suggesting the potential role of BChE in cholinergic and PSNS function in HFD mice, we knowledge that there are some obvious limitations of this study that need to be further addressed in the future. First, the present study only tested the vagal control of the heart rate in anesthetized animals. It is important to test this altered vagal response in conscious HFD mice. Second, the role of the enhanced BChE in cholinergic function needs to be further tested using genetic approaches. Third, acute vagal stimulation in this study revealed a clear attenuation of the response in heart rate in HFD mice. However, this observation cannot determine whether the basal level of vagal control of the heart or “vagal tone” is altered with HFD. In addition, further investigations of BChE expression and functional roles in cholinergic transmission in the central and other peripheral tissues of this HFD model will also be interesting and are important for better understanding how this enzyme regulates cholinergic synaptic activities as well as its potential therapeutic target in abnormal health conditions. Moreover, it would be also interesting and important to determine the factors that may cause the upregulation of BChE in obesity. In our model, blood glucose and cholesterol levels were all significantly increased. Whether hyperglycemia and/or hypercholesterolemia can increase BChE level remains to be tested. Although the plasma level of resistin, a major pathological factor in obesity, metabolic disorders, and diabetes, was unchanged in our model, the role of other adipokines and cytokines remain to be further studied. It should be noted that there are more molecules and complex mechanisms that are also involved in peripheral PSNS dysfunction. For example, the role of nicotinic receptors in cardiac parasympathetic ganglionic transmission has been found to be involved in PSNS dysfunction in chronic heart failure (Bibevski and Dunlap 1999, 2004). Nitric oxide has been found as an important modulator in Ach release in cardiac parasympathetic terminals (Mohan et al. 2002; Chowdhary et al. 2004). The impaired PSNS function could well be a consequence of multiple factors. Nonetheless, this study provides a novel piece of evidence that increased BChE may be one of contributors to peripheral PSNS dysfunction and thus a potential therapeutic target.

## Conflict of Interest

None declared.
